# Theoretical Study of Proton Tunneling in the Imidazole–Imidazolium Complex

**DOI:** 10.1021/acs.jpca.1c02972

**Published:** 2021-08-05

**Authors:** Łukasz Boda, Marek Boczar, Marek J. Wójcik, Takahito Nakajima

**Affiliations:** † Faculty of Chemistry, Jagiellonian University, Gronostajowa 2, 30-387 Kraków, Poland; ‡ RIKEN, Center for Computational Science, 7-1-26, Minatojima-minami-machi, Chuo-ku, Kobe, Hyogo 650-0047, Japan

## Abstract

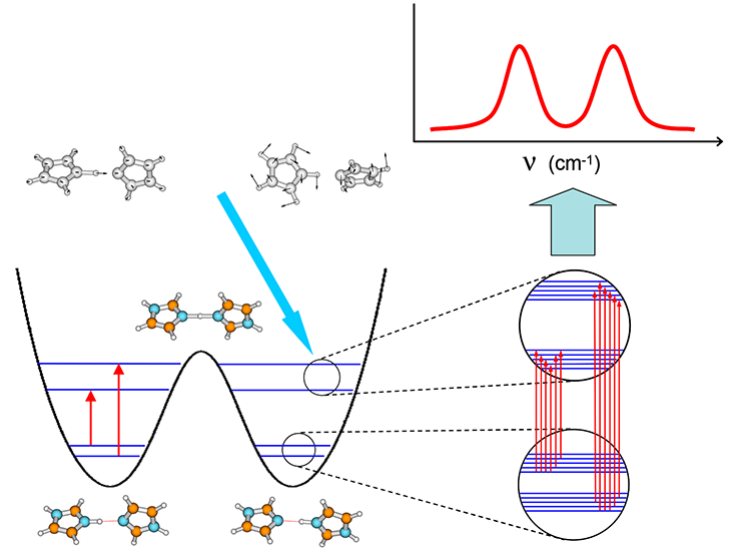

Proton tunneling in the hydrogen-bonded imidazole–imidazolium complex ion has been studied theoretically. *Ab initio* CASSCF/6-311++G(d,p) calculations concerning geometry optimization and vibrational frequencies have been carried out for equilibrium and transition state structures of the system. Two-dimensional double-well model potentials were constructed on the basis of *ab initio* results and used to analyze the proton dynamics in the hydrogen bond and the influence of the excitation of low-frequency hydrogen-bond vibrations on the proton tunneling splittings. The energy of tunneling-split vibrational sublevels of the high-frequency tunneling mode have been calculated for its ground and first excited vibrational state for the series of excitations of the coupled low-frequency intramolecular hydrogen-bond modes. The promoting and suppressing effect of the low-frequency modes on the proton splittings was shown in the ground and first excited vibrational state of the tunneling mode. The vibrational sublevels form the two separate semicontinuous bands between which the absorption transitions may occur. This mechanism explains the experimentally observed splitting and doublet-component broadening of the high-frequency N–H stretching infrared (IR) absorption band.

## I Introduction

The tunneling process is of great importance in many areas of physics, chemistry, and biology.^1−3^ The tunneling phenomenon is also one of the most significant features of quantum mechanics, completely different from the classical one. In a variety of tunneling processes, proton-transfer (PT) reactions are of special interest, especially in systems with hydrogen bonds. The proton tunneling in hydrogen-bonded systems is an important and fundamental process in nature, especially in the biological aspect, e.g., for DNA base pairing.^4^ In the hydrogen bond, the hydrogen atom is shared between the donor and acceptor, which may result in classical proton transfer or tunneling, depending on the strength of the hydrogen bond. Additionally, proton dynamics in the double-well potential can be affected by strong interactions with vibrating surrounding atoms within a hydrogen-bonded system. Infrared (IR) spectroscopy can provide key information on the tunneling dynamics and influence of vibrational couplings in a hydrogen-bonded complex on this process. In the last years there appeared several theoretical studies of proton tunneling in different systems,^5−19^ as well as experimental ones.^20−24^

Theoretical studies of proton tunneling usually are based on multidimensional potential energy surfaces that sometimes are not easy to obtain from *ab initio* calculations, especially for electronically excited states. Proton tunneling in the ground electronic state is more easily tractable.

In this article, we focus on the proton dynamics in the ground electronic state of an intermolecular hydrogen-bonded complex—the cation of imidazole (Im) with protonated imidazole (imidazolium, ImH^+^). Inspiration for this work was the experimental study of Bonsor et al.^25^ In this work concerning complex hydrogen-bonded cations, the authors have recorded the IR absorption spectra for solutions of ImH^+^ salts with several anions and evidenced the existence of two separate broad bands related to the N–H stretching for the solution of imidazolium salt with perchlorate anion for the concentrations in which the (Im)_2_H^+^ complex cation has been formed. On the other hand, no doubling of the N–H stretching band was observed in IR spectra of solutions as well as solid ImH^+^ClO_4_^–^, where the complex cation (Im)_2_H^+^ was not present. The authors interpreted the observed doublet as an effect of a double-minimum potential for hydrogen involved in the intermolecular hydrogen bond and associated proton tunneling process. The aim of this work is to explain this process in a theoretical way, assuming that the proton tunneling process is responsible for the doublet observed in the IR spectra of the ImH^+^–Im complex.

The imidazole–imidazolium complex cation has been the subject of previous theoretical studies. Yan et al.^26^ studied the coupling character between Im and ImH^+^ and its implication for the coupling modes of biomolecular residues. On the basis of the density functional theory (DFT)-calculated global potential energy surface the authors have found 19 stable structures of the ImH^+^–Im complex and four kinds of coupling modes. Tatara et al.^27^ studied the potential energy surfaces for proton transfer between the Im and ImH^+^ moieties and for rotation of the Im and ImH^+^ moieties around the hydrogen bond in the ImH^+^–Im complex. This work is a continuation of this study and also another of our studies concerning proton tunneling in hydrogen-bonded systems.^28−30^

This paper is organized as follows. The results of our *ab initio* calculations for the ImH^+^–Im complex cation are presented in section II. The model used for calculation of tunneling splittings in the ImH^+^–Im complex is presented in section III. The results are presented and discussed in relation to the experimentally observed IR band doubling in section IV. Concluding remarks are presented in section V.

## II Quantum Chemical Calculations

*Ab initio* studies of proton-transfer processes require proper treatment of an electronic structure in order to capture subtleties of the potential energy surface. The existence of the intermolecular hydrogen bond, being a noncovalent interaction, as well as taking into account the proton transfer, being in fact the bond rearrangements process, requires the multiconfigurational character of the electronic wave function. For this reason, for study of the ImH^+^–Im complex we employed the complete active space self-consistent (CASSCF) method.^31^ An essential feature of the CASSCF calculation is the choice of a judicious set of occupied and virtual orbitals forming the so-called active space. Within the active space a full configuration interaction (CI) is performed, with the remaining orbitals (not included in the active space) being doubly occupied in all configurations. For proper description of the electronic structure of the ImH^+^–Im complex we took into account in the active space all π orbitals (occupied π and virtual π*) of the imidazole rings, the lone-pair orbital *n*, and a pair of σ and σ* orbitals localized on the N–H···N hydrogen bond as being relevant for correct description of the proton-transfer process. This gives an active space of (16,13), i.e., composed of 16 electrons and 13 orbitals. Geometry optimizations and normal-mode analysis were performed at the CASSCF(16,13) level with the triple-ζ valence 6-311G-type Gaussian basis, added with the set of diffuse and polarization functions for all atoms, to give the 6-311++G(d,p) basis set.^32^ To check the correlation effects (dynamical correlation) beyond this approximation we employed second-order multireference perturbation theory (CASPT2) with respect to the CASSCF reference. Only single-point energy calculations have been performed for the equilibrium and saddle point structures at the CASPT2/6-311++G(d,p) level as the analytical gradients are presently unavailable for the internally contracted variant of CASPT2 method (RS2C in MOLPRO),^33^ which was chosen due to its greater efficiency for larger active space reference wave functions. CASSCF/CASPT2 calculations have been carried out using the MOLPRO program package^34,35^ using tight convergence criteria (compared to the defaults). In order to evaluate the stability of the ImH^+^–Im complex in water solution and estimate qualitatively its formation energy, we performed DFT calculations for geometry optimization for the complex and for the separate Im and ImH^+^ moieties in the presence of water solvent using the Gaussian 09 program package.^36^ The self-consistent reaction field (SCRF) with the polarizable continuum model (PCM)^37^ has been chosen to perform the calculations in the presence of a water solvent together with the CAM-B3LYP hybrid exchange-correlation functional,^38^ being a long-range-corrected version of the B3LYP functional, as more adequate for the description of a hydrogen-bonded system in polar solvent and the 6-311++G(d,p) basis set. All calculations have been run on a Dell Precision T7500 workstation equipped with two hexacore Intel Xeon X5680 processors and 192 GB of RAM memory.

**Figure 1:**
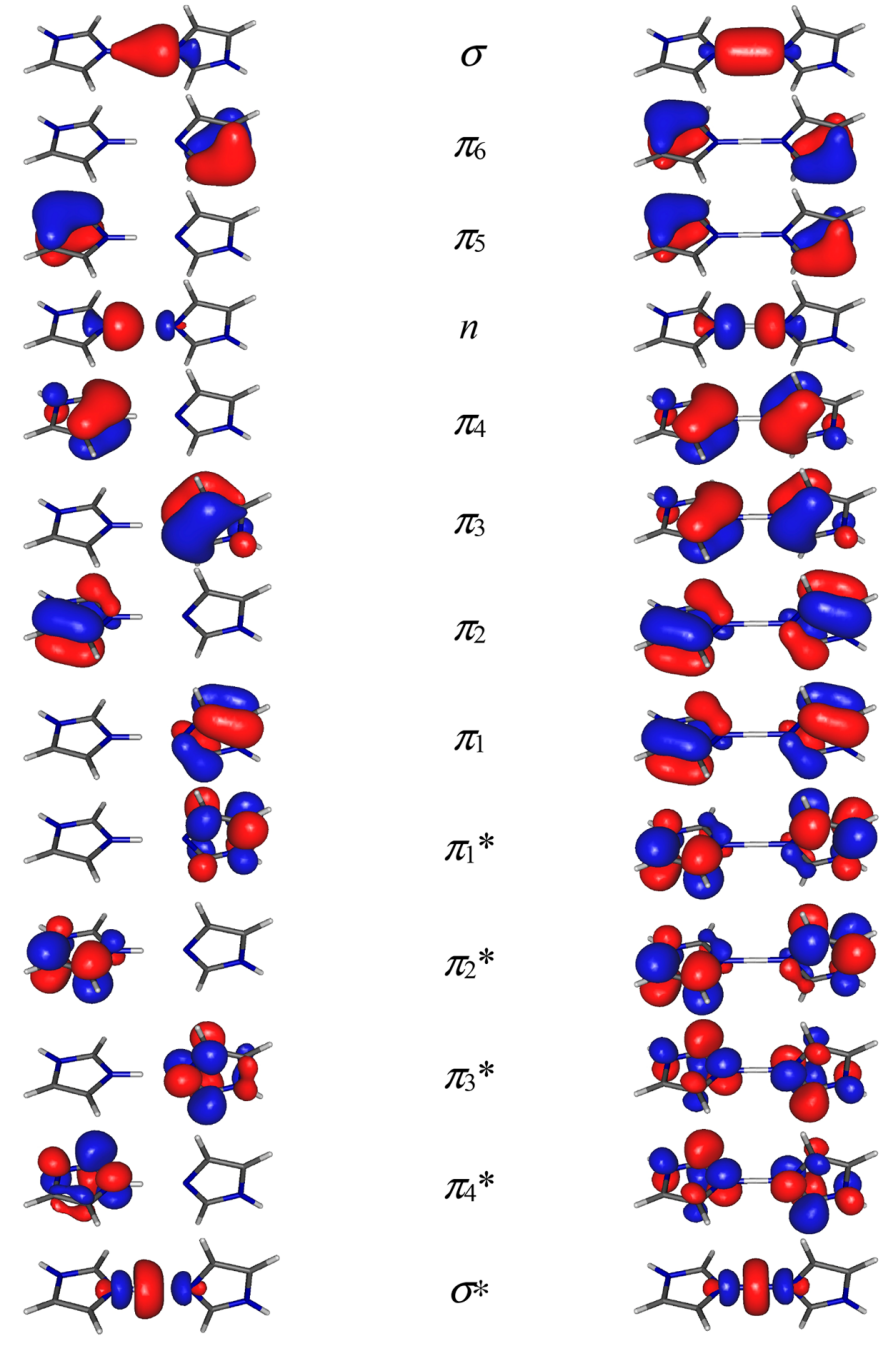
CASSCF active space orbitals of the ImH^+^–Im hydrogen-bonded complex cation determined for the equilibrium (left) and transition state geometry (right).

**Figure 2:**
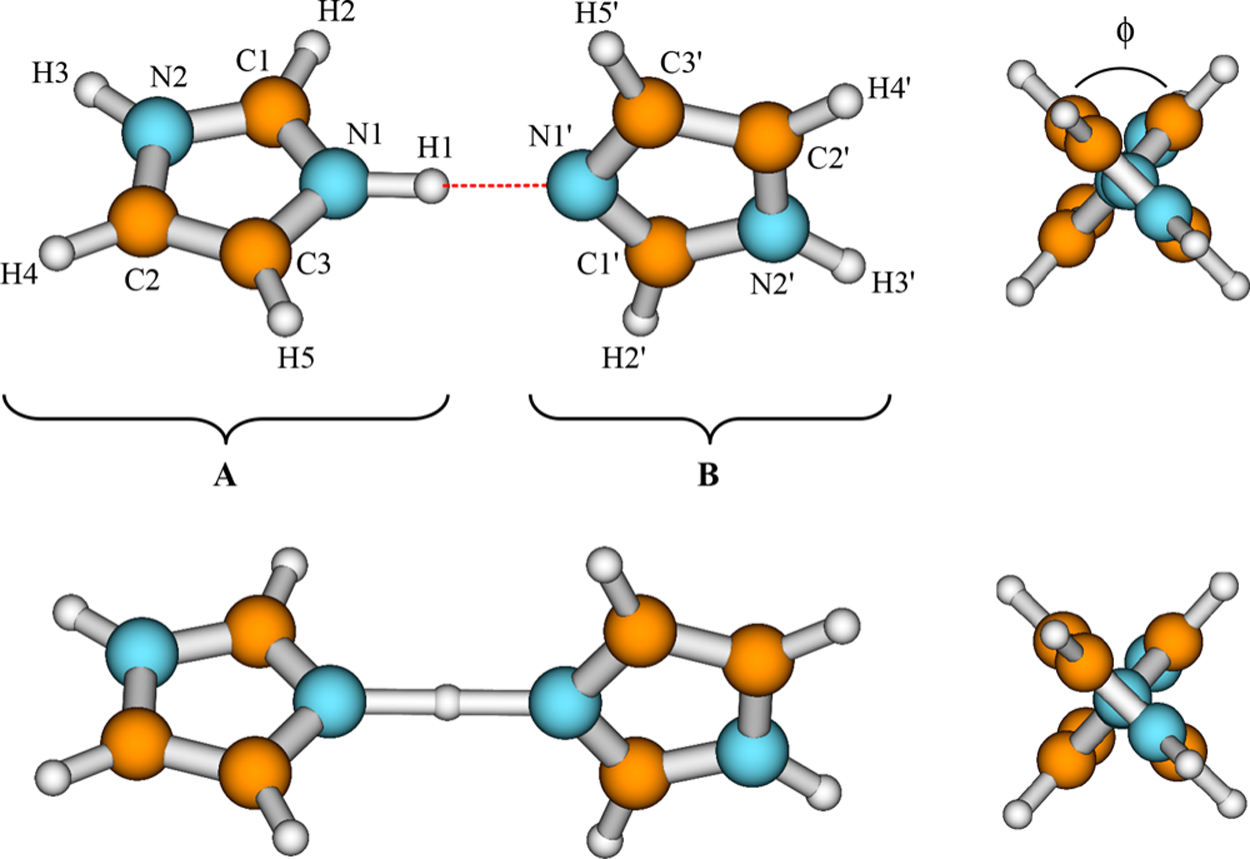
Structures of the imidazole–imidazolium (ImH^+^–Im) complex in the stable point (top) and saddle point (bottom) optimized at the CASSCF(16,13)/6-311++G(d,p) level, with numbering of atoms and labeling of the imidazole rings.

The active space orbitals at the stable and transition state geometry obtained from CASSCF(16,13)/6-311++G(d,p) calculations are presented in Figure 1. They have been chosen on the basis of canonical Hartree–Fock orbitals. The optimized geometries of the stable and saddle point structures for proton transfer are presented in Figure 2. The bond lengths and bond angles for both structures are listed in Table 1 together with the available diffraction data obtained for the crystal of imidazolium–imidazole perchlorate,^39^ the same compound that was studied by IR spectroscopy in the solid state and in solution.^25^

**Table 1:** Optimized Geometries of the Stable and Saddle Point Structures of the ImH^+^–Im Hydrogen-Bonded Complex by the CASSCF(16,13)/6-311++G(d,p) Method

	calcd		exptl
	stable structure	saddle point structure	(Im)_2_H^+^ClO_4_^–^ crystal^a^
Bond Lengths (Å)			
N1···N1′	2.760	2.581	2.73
H1···N1′	1.687	1.291	
N1–H1	1.073	1.291	
N1–C1	1.311	1.309	1.311
C1–N2	1.327	1.335	1.293
N2–C2	1.379	1.376	1.385
C2–C3	1.356	1.358	1.323
C3–N1	1.379	1.380	1.370
C1–H2	1.069	1.070	
N2–H3	0.996	0.995	
C2–H4	1.067	1.068	
C3–H5	1.067	1.068	
N1′–C1′	1.307	1.309	1.296
C1′–N2′	1.343	1.335	1.334
N2′–C2′	1.373	1.376	1.335
C2′–C3′	1.359	1.358	1.335
C3′–N1′	1.382	1.380	1.396
C1′–H2′	1.070	1.070	
N2′–H3′	0.994	0.995	
C2′–H4′	1.069	1.068	
C3′–H5′	1.069	1.068	
Angles (deg)			
N1–H1–N1′	178.4	179.2	
C1–N1–H1	124.5	126.1	
N1–C1–N2	108.8	110.1	108.6
C1–N2–C2	109.0	108.4	107.9
N2–C2–C3	106.2	105.8	106.6
C2–C3–N1	107.0	108.3	108.2
C3–N1–C1	109.0	107.4	108.5
C3–N1–H1	126.5	126.5	
N1–C1–H2	125.7	125.7	
C1–N2–H3	125.0	125.4	
N2–C2–H4	122.4	122.5	
C2–C3–H5	130.8	129.6	
C1′–N1′–H1	128.8	126.1	
N1′–C1′–N2′	111.3	110.1	113.0
C1′–N2′–C2′	107.8	108.4	108.4
N2′–C2′–C3′	105.5	105.8	106.2
C2′–C3′–N1′	109.4	108.3	108.8
C3′–N1′–C1′	106.0	107.4	103.6
C3′–N1′–H1′	125.2	126.5	
N1′–C1′–H2′	125.7	125.7	
C1′–N2′–H3′	125.8	125.4	
N2′–C2′–H4′	125.6	122.5	
C2′–C3′–H5′	128.5	129.6	

a Ref (24).

The stable structure of the complex has *C*_1_ symmetry. Both imidazole rings are almost planar, with a maximum displacement from the least-squares plane of 0.021 Å which agrees very well with the experimental value 0.015 Å^39^ and with previous calculations.^27^ The imidazole rings are linked together by an intermolecular symmetric (N···N-type) hydrogen bond which is almost linear (178.4° N–H···N angle). This hydrogen-bond length is 2.760 Å; the N–H length is 1.073 Å. The planes of the imidazole rings are rotated one to another by an angle ϕ (see Figure 2). We performed relaxed potential energy surface (PES) scan calculations for the rotation of imidazolium about the N–H···N axis within the range of ϕ from 0° to 360° with a 5° step. The obtained PES revealed the two equivalent minima at ca. 90° and ca. 270° for the ϕ angle. The experimental value is ca. 50° which is in significant discrepancy with the result of calculation. The discrepancy may result from interactions in the crystal lattice, especially from the weaker hydrogen bonds between the ImH^+^–Im units and their counterparts in adjacent cells via oxygens of the perchlorate, whereas the calculations have been done for the isolated ImH^+^–Im complex cation. The calculated bond lengths and bond angles are in good agreement with crystallographic data. In the saddle point structure the proton in the hydrogen bond lies exactly in the middle of the N···N distance. As compared with the stable structure, we observe a significant increase of the N–H length to the value of 1.281 Å together with the shortening in the N···N hydrogen-bond length to 2.581 Å. The planarity of the imidazole rings in the saddle point is higher (0.008 Å is the maximum displacement from the least-squares plane), and the planes are close to perpendicular orientation (ϕ = 88.6°). Bond lengths and bond angles for rings A and B (primed and not-primed in Figure 2) are equal giving the *C*_2_ symmetry for the saddle point structure, where the twofold axis lies on a bisector of angle ϕ passing through the H1 hydrogen. After complete proton transfer from ring A to B an equivalent structure will be formed. We also estimated the energetics of the ImH^+^–Im complex and free ImH^+^ and Im molecules in the presence of water solvent through the DFT SCRF calculations. The SCRF CAM-B3LYP-calculated absolute bonding energy obtained by the supermolecule approach and taken as the difference of the total electronic energy of the ImH^+^–Im complex and the total electronic energy of the free ImH^+^ and Im monomers is −0.01721 au (45.18 kJ/mol), which indicates the favoring of complexation compared to the existence of free monomers and suggests the stability of the complex in aqueous solution. It should be mentioned that the calculated value of the binding energy of the complex may be overestimated, mainly due to the BSSE (basis set superposition error), as the counterpoise correction was unavailable for the performed SCRF calculations. No significant changes in geometry are observed in the SCRF CAM-B3LYP/6-311++G(d,p)-optimized structure of the ImH^+^–Im complex compared to that optimized in the vacuum calculation at the CASSCF/6-311++G(d,p) level. The SCRF-optimized N1···N1′ hydrogen-bond length is 2.724 Å, the N1–H1 bond length is 1.074 Å, and the ϕ angle is 88.8°.

Table 2 contains harmonic frequencies of the normal modes of the ImH^+^–Im complex in the stable and saddle point structures calculated by the CASSCF method. The approximate description of the normal modes concerns mainly the stable structure; however, the frequencies for the saddle point structure have been matched to their analogues for the stable structure and properly sorted in Table 2. The description of the normal modes for the saddle point structure is analogous to that of the stable one but without distinguishing the amplitudes between the two moieties of the complex due to the degeneracy of the normal modes for the saddle point structure as an effect of its higher symmetry (*C*_2_) compared to that of the stable one (*C*_1_). The formation of the ImH^+^–Im complex strongly influences some existing vibrational frequencies and creates new vibrational degrees of freedom, related to the hydrogen bond, as compared to the ones for the free Im and ImH^+^ molecules. Due to our interest in proton dynamics in the hydrogen bond and in modeling proton tunneling, the normal modes within the hydrogen bond are of special importance. These are three intermolecular low-frequency vibrations: the hydrogen-bond stretching mode, labeled ν_σ_, the two low-frequency mutually perpendicular hydrogen-bond bending modes, labeled ν_β1_ and ν_β2_, and the high-frequency N1–H1 stretching mode (tunneling mode), labeled ν_s_. The N1–H1 stretching mode has a significantly decreased frequency in the ImH^+^–Im complex compared to this frequency in the free ImH^+^ molecule^27^ and to the frequency of the N2–H3 and N2′–H3′ stretching modes in the ImH^+^–Im, because these modes do not form hydrogen bonds. The calculated N1–H1 stretching mode (ν_s_) frequency is 2385 cm^–1^ as compared to 2049 cm^–1^ in the previous study of Tatara et al.,^27^ obtained at the B3LYP/6-311++G(d,p) level. Table 2 also contains the calculated frequencies of the ImH^+^–Im in its transition state (saddle point structure). The only one imaginary frequency (991*i* cm^–1^) is related to the ν_s_ mode which shifts the proton from the center of the ImH^+^–Im molecule toward the left or right imidazole ring. The experimental frequency of the hydrogen-bonded N–H stretching was obtained by Bonsor et al.^25^ The authors have recorded the IR absorption spectra of the ImH^+^–Im complex in the crystal and in solution and observed two separate broad sub-bands originating from hydrogen-bonded N–H stretching and centered at ca. 1900 and ca. 2500 cm^–1^ in the crystal and at 1950 and ca. 2500 cm^–1^ in solution. The authors suggested that the proton tunneling phenomenon associated with a double-well minimum potential could give rise to the ν_s_ band doublet, in both the crystal and solution spectra. They also confirmed that, after allowance for specific bands, the spectra in solution are independent to the counterion or the solvent used. The theoretical reconstitution of the ν_s_ band splitting related to the proton tunneling process is the main aim of this work.

**Table 2:** Calculated Harmonic Vibrational Frequencies for the Stable and Saddle Point Structures of the ImH^+^–Im Complex^a^

	stable structure		saddle point structure
no.	ν [cm^–1^]	approximate description^b^	ν [cm^–1^]
1	29	monomers twisting (torsion)	32
2	49	H-bond bending (ν_β1_)	61 (ν_β1_)
3	51	H-bond bending (ν_β2_)	62 (ν_β2_)
4	128	monomers rocking	165
5	141	N···N H-bond stretching (ν_σ_)	265 (ν_σ_)
6	152	monomers rocking	165
7	585	γ(N2′–H3′)	614
8	632	oop ring (A) deform	664
9	654	oop ring (A) deform	664
10	663	oop ring (B) deform	703
11	705	oop ring (B) deform	703
12	708	γ (N2–H3)	615
13	744	γ (C–H) ring (B)	747
14	747	γ (C–H) ring (A)	747
15	864	γ (C–H) ring (B)	869
16	868	γ (C–H) ring (A)	869
17	886	γ (C–H) ring (B)	912
18	926	γ (C–H) ring (A)	912
19	952	ip rings (A) + (B) deform	994
20	991	ip rings (A) + (B) deform	996
21	996	ip rings (A) + (B) deform	1047
22	1016	ip rings (A) + (B) deform	1049
23	1133	ν(C–N) ring (A)	1145
24	1150	ν(C–N) ring (B)	1151
25	1176	ν(C–N) ring (A)	1187
26	1182	ν(C–N) ring (B)	1194
27	1188	ν(C–C) + δ(C–H) ring (B)	1206
28	1195	ν(C–C) + δ(C–H) ring (A)	1212
29	1246	γ(N1–H1) + ring (B) breathing	1274
30	1255	δ(N2′–H3′) + ring (B) breathing	1286
31	1271	δ(N2–H3) + ring (A) breathing	1366
32	1337	δ(C–H) ring (A)	1376
33	1392	δ(C–H) ring (B)	1451
34	1443	δ(C–H) ring (A)	1452
35	1464	δ(C–H) ring (B)	1586
36	1582	δ(N2′–H3′)	1620
37	1589	δ(N2–H3)	1587
38	1629	δ(N1–H1) + δ(N2–H3)	1621
39	1653	δ(N2′–H3′)	1587
40	1664	δ(N2′–H3′) + δ(C–H) ring (B)	1674
41	1697	δ(N2–H3) + δ(C–H) ring (A)	1675
42	1754	δ(N1–H1) + δ(N2–H3) + δ(C–H) ring (A)	1724
43	2315	ν(N1–H1) (ν_s_)	991*i*(ν_s_)
44	3208	ν(C–H) ring (B)	3223
45	3211	ν(C–H) ring (B)	3223
46	3233	ν(C–H) ring (A)	3224
47	3234	ν(C–H) ring (A)	3224
48	3238	ν(C–H) ring (B)	3245
49	3253	ν(C–H) ring (A)	3245
50	3659	ν(N2–H3)	3672
51	3684	ν(N2′–H3′)	3673

a ν, stretching; δ, in-plane bending; γ, out-of-plane bending; τ, torsion.

b ip and oop mean in-plane and out-of-plane, respectively, and together with the δ and γ labels refer to the local plane of each imidazole ring of the complex.

## III Model for Proton Tunneling

In order to obtain quantitative results on the structure of the vibrational levels and absorption transitions in the doublet in the IR spectra of ImH^+^–Im, we take into account the dynamics of the proton in the double-well minimum potential. Our consideration is confined to the tunneling in the two-dimensional symmetric double-well potential (SDWP).^40,41^

The following Hamiltonian is assumed:
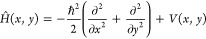
1where *x* and *y* denote the mass-scaled coordinates.

**Figure 3:**
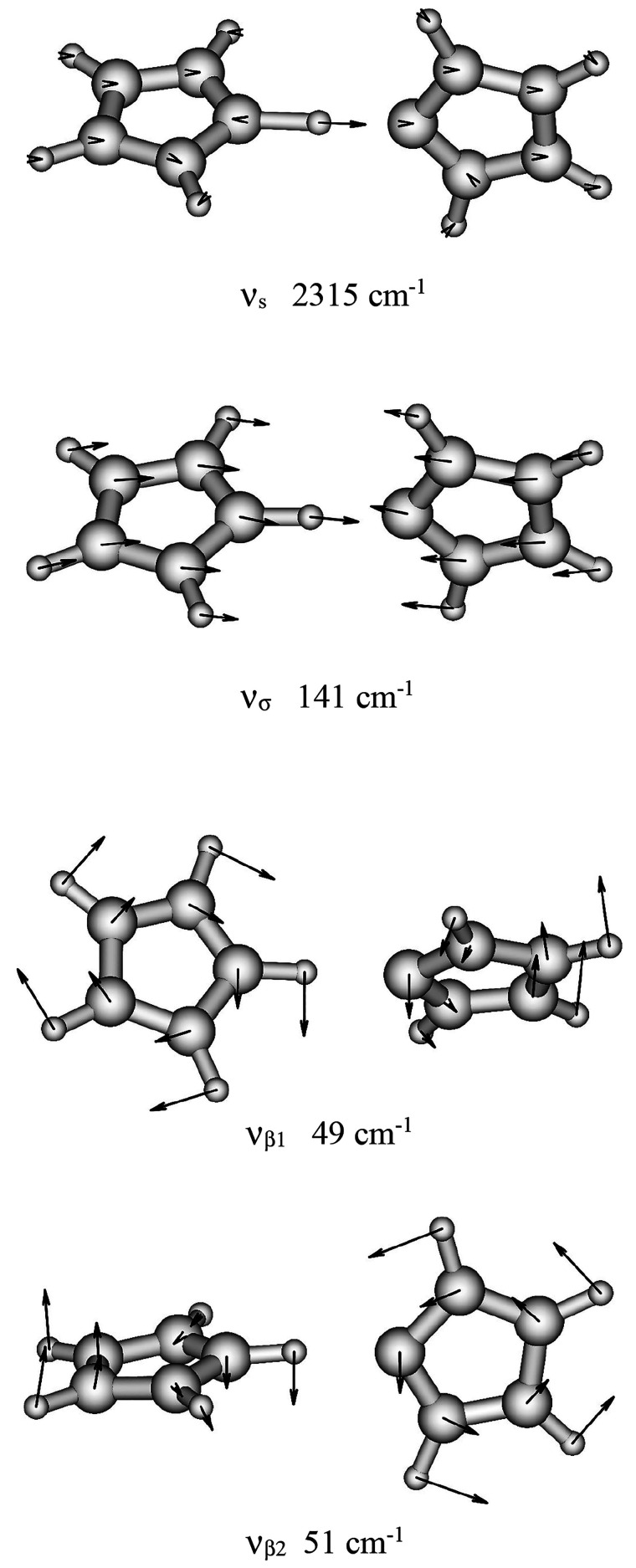
Selected normal modes of the ImH^+^–Im complex calculated by the CASSCF(16,13)/6-311++G(d,p) method.

In order to calculate the splittings of the vibrational energy levels for ν_s_ (mode no. 43 in Table 2), we constructed, on the basis of *ab initio* calculations, two-dimensional model potentials for the proton tunneling mode ν_s_ coupled to selected low-frequency modes which strongly affect the tunneling. These are an intermolecular hydrogen-bond stretching mode ν_σ_ (no. 5 in Table 2) and the low-frequency hydrogen-bond bending modes ν_β1_ and ν_β2_ (nos. 2 and 3 in Table 2). They are presented together with the tunneling mode in Figure 3. The mode ν_σ_ occurs along the axis of the N–H···N hydrogen bond and is supposed to promote the tunneling, whereas the two bending modes ν_β1_ and ν_β2_ are approximately perpendicular to each other and occur out of axis of the hydrogen bond. They are supposed to suppress the tunneling.

Let us denote by ω_*x*_ the frequency of the tunneling mode ν_s_ and by ω_*y*_ one of the frequencies of the modes ν_σ_, ν_β1_, or ν_β2_. To consider the Schrödinger equation with the Hamiltonian (eq 1) in SDWP, we assume an adiabatic approximation for the wave functions representing the tunneling mode and the low-frequency mode, as the frequency ω_*y*_ is much lower than ω_*x*_. Within this assumption the total wave functions can be written as^41^
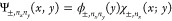
2where ± specifies the parity with respect to the *y* axis; the adiabatic bases χ_±,*n*_*x*__ and the coefficient functions ϕ_±,*n*_*x*_*n*_*y*__ are defined by the following eigenvalue equations:
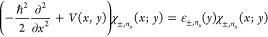
3
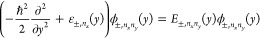
4Since ϕ is not directly related to tunneling, eq 4 may be approximated by the separable equation:
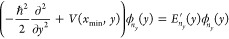
5where *x*_min_ is the *x* coordinate of the potential minimum. The localized wave functions Ψ^L^ and Ψ^R^ can be obtained by taking symmetric and antisymmetric linear combinations of Ψ_±,*n*_*x*_*n*_*y*__, i.e.:
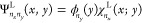
6and
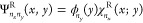
7The final expression for energy splitting can be obtained as
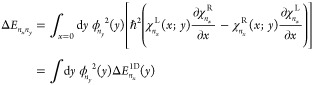
8where Δ*E*_*n*_*x*__^1D^(*y*) is the one-dimensional expression of energy splitting.

The physical meaning of eq 8 is similar to the Franck–Condon principle and can be stated as follows: the tunneling in the *x* direction occurs at a fixed value of *y*, causing the energy splitting Δ*E*_*n*_*x*__^1D^(*y*), and the total energy splitting can be obtained by averaging these values over *y* with weight factors ϕ_*n*_*y*__^2^.

From several typical model potentials introduced to interpret the mechanisms of promotion and suppression of tunneling by the vibrational excitation in the mode coupled to the tunneling one, we have chosen the following two proposed by Takada and Nakamura:^41^

(a) the symmetric mode coupling potential (SMC) describing couplings of the proton tunneling mode ν_s_ with the hydrogen-bond coaxial mode ν_σ_
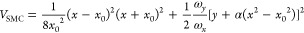
9and

(b) the squeezed double-well potential (SQZ) describing couplings of the proton tunneling mode ν_s_ with the hydrogen-bond bending modes ν_β1_ and ν_β2_
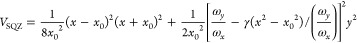
10where *x* and *y* denote the coordinates of the proton tunneling and the low-frequency modes, respectively, ω_*x*_ and ω_*y*_ are the angular frequencies, 2*x*_0_ is the distance between the two minima, and α and γ are the parameters describing coupling strength. In formulas 9 and 10, the potentials are expressed in the units of the quantum ℏω_*x*_ and the coordinates *x* and *y* are dimensionless via the following formulas:
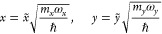
11*x̃* and *ỹ* denote the dimensional coordinates, and *m*_*x*_ and *m*_*y*_ are the effective masses. The parameters *x*_0_, α, and γ of potentials 9 and 10 can be estimated from the following formulas:
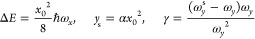
12where Δ*E*, *y*_s_, and ω_*y*_^s^ denote the energy barrier, the value of the normal coordinate *y* of the coupled low-frequency mode for the SMC potential at the saddle point, and the angular frequency of the mode *y* at the saddle point structure for the SQZ potential, respectively. The choice of the low-frequency modes which promote or suppress the tunneling was done on the basis of their symmetry and amplitudes, and therefore, the coordinate *x* in potential eq 9 mainly represents the N–H stretching motion (ν_s_ mode) of the hydrogen atom transferring from N to N, while *y* represents the N···N stretching motion (ν_σ_ mode), since the change of the N···N hydrogen-bond distance influences the probability of tunneling. Similarly, in potential eq 10 the *y* coordinate corresponds to the N–H···N hydrogen-bond bending motion, and since in the equilibrium structure of the complex the hydrogen bond is linear, an out-of-hydrogen-bond-line deviation changes the N···N distance. The remaining low-frequency modes do not change the N···N distance and are assumed not to be affecting the tunneling.

## IV Results and Discussion

The values of the energy and energy barrier height for proton transfer in the ImH^+^–Im complex, resulting from *ab initio* CASSCF calculations, and essential in determining the parameters of the model potentials, are summarized in Table 3. The energy barrier is relatively high (28.95 kJ/mol), and its value changes when the zero-point energy (ZPE) correction is taken into account. There are three slightly different energy barrier values obtained by including ZPE only for vibrational degrees of freedom other than the two degrees of freedom (the high-frequency tunneling mode and low-frequency promoting/suppressing mode) that are treated explicitly in the model calculations based on two-dimensional potentials constructed for the three pairs of modes, i.e., ν_s_–ν_σ_, ν_s_–ν_β1_, and ν_s_–ν_β2_. It should be mentioned that the energy barrier strongly decreases when the ZPE correction is taken into account for all vibrational degrees of freedom and has the value 18.97 kJ/mol (1586 cm^–1^). On the other hand all calculated values are lower than the value of 38 kJ/mol obtained by Basch et al.;^42^ however, no complete geometry was performed in that study. Correlation energy corrections to the energies for the CASSCF-optimized equilibrium and saddle point structures of ImH^+^–Im, obtained at the CASPT2 level, have the values of −1.099764 and −1.100353 au, respectively, which gives the difference of 0.000589 au (129 cm^–1^). Such value is about 5% of the total energy of the barrier (2420 cm^–1^ obtained at the CASSF level without ZPE correction), which can be considered as giving rise to a negligible error.

**Table 3:** Calculated Energies and Proton-Transfer Energy Barriers of the ImH^+^–Im Complex

	no ZPE	with ZPE (excluding ν_s_, ν_σ_)	with ZPE (excluding ν_s_, ν_β1_)	with ZPE (excluding ν_s_, ν_β2_)
Energy				
stable structure (au)	–451.293899	–451.134681	–451.134471	–451.134476
saddle point structure (au)	–451.282871	–451.122463	–451.121998	–451.122010
Energy Barrier				
(kJ/mol)	28.95	32.06	32.75	32.73
(cm^–1^)	2420	2680	2737	2735

**Figure 4:**
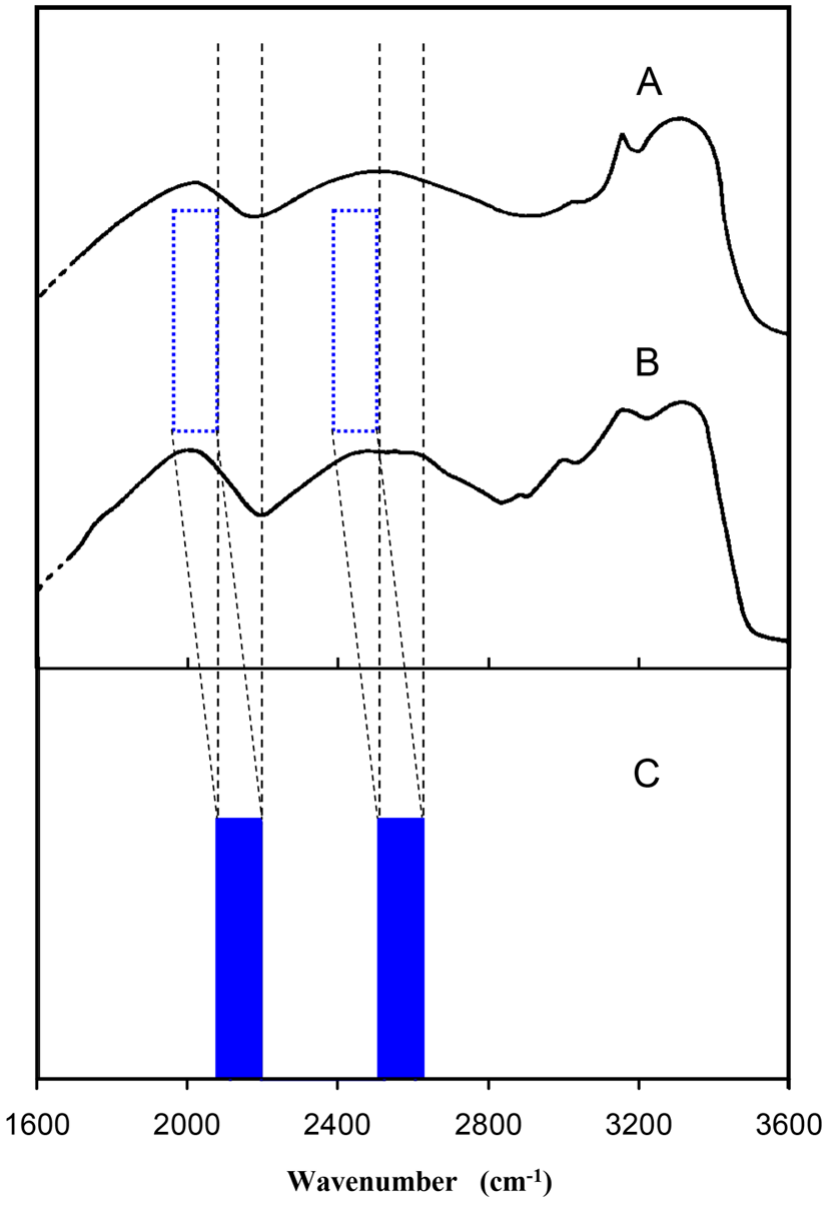
Infrared spectra in the NH stretching range. (A) solid (Im)_2_H^+^ClO_4_^–^, (B) ImH^+^ClO_4_^–^ + Im, in 2 M solution. Solvent and counterion bands have been omitted. (Adapted with permission from ref (25). Copyright 1976 Canadian Science Publishing.) (C) Theoretical reconstitution of the N–H stretching band doublet as an effect of proton tunneling.

On the basis of the results obtained through *ab initio* calculations and using the ZPE-corrected energy barriers from Table 3, we determined the parameters of the model SMC and SQZ potentials. Their values used in subsequent calculations are listed in Table 4. It should be mentioned that model potential eqs 9 and 10 describe the low-frequency modes (*y* coordinate) in the harmonic approximation. Such vibrations usually reveal strong anharmonicity. However, harmonic treatment of these modes has small influence on the obtained splitting values, due to the small values of the coupling parameters α and γ in potential eqs 9 and 10, which allows us to avoid the use of complex forms of the two-dimensional anharmonic potentials, which are inconvenient for numerical calculations. Tunneling energy splittings have been calculated by a variational procedure applied to eq 1 with the parametrized SMC or SQZ potential and, subsequently, by the DVR (discrete variable representation) method.^43,44^ The resulting tunneling splittings of the vibrational states of the ν_s_ mode in its ground (*n*_*x*_ = 0) and first excited state (*n*_*x*_ = 1) for the series of excitations of low-frequency modes ν_σ_, ν_β1_, and ν_β2_ (*n*_*y*_ = 0, 1, 2, ...) are presented in Table 5. It can be observed that the applied two-dimensional model potentials fitted to the quantities obtained from *ab initio* calculations give tunneling splittings Δ*E*_0_ for the ground (*n*_*x*_ = 0) and Δ*E*_1_ for the first excited state (*n*_*x*_ = 1) of the ν_s_ mode (high-frequency N1–H1 stretching) and their dependence on vibrational excitations of low-frequency modes. The splitting values Δ*E*_1_ for the *n*_*x*_ = 1 level are much higher than the values Δ*E*_0_ for the *n*_*x*_ = 0 level. A long sequence of monotonic increase of the energy splittings Δ*E*_0_ and Δ*E*_1_ accompanying excitations of the ν_σ_ mode and monotonic decrease of Δ*E*_0_ and Δ*E*_1_ accompanying excitations of the ν_β1_ and ν_β2_ modes can be observed. Such a mechanism introduces a complex structure of vibrational sublevels for the ν_s_ mode, between which absorption transitions can occur. For a fixed value of quantum number *n*_*y*_, the vibrational level *n*_*x*_ = 0 is split into two sublevels *E*_0_^+^ and *E*_0_^–^ with an energy gap of Δ*E*_0_ = *E*_0_^–^ – *E*_0_^+^, and analogously, *n*_*x*_ = 1 is split into two levels *E*_1_^+^ and *E*_1_^–^ with an energy gap of Δ*E*_1_ = *E*_1_^–^ – *E*_1_^+^. Under the *G*_4_ molecular symmetry group only the two absorption transitions between *E*_0_^+^, *E*_0_^–^, *E*_1_^+^, and *E*_1_^–^ are allowed.^45^ These are the higher-energy transition T1, *E*_1_^+^ → *E*_1_^–^, and lower-energy transition T2, *E*_0_^–^ → *E*_1_^+^. The energy of T1 is a sum of the energy of T2 and the tunneling splittings for both the ground and first excited states of the ν_s_ mode, or in other words, the difference Δ_T1–T2_ between the energy of transitions T1 and T2 is a sum of the tunneling splittings Δ*E*_0_ and Δ*E*_1_. The value of Δ_T1–T2_ is also equal to the spacing between the two components of the ν_s_ doublet observed in the IR absorption spectrum. As can be seen from Table 5, the values of Δ*E*_0_ and Δ*E*_1_ range from 67.0 to 129.2 cm^–1^ and from 250.5 to 403.6 cm^–1^, respectively; consequently, the value of Δ_T1–T2_ changes from ca. 317 to 533 cm^–1^ contributing to the partial broadening of the doublet components. The theoretically predicted absorption doublet concerning the ν_s_ mode of the ImH^+^–Im complex is presented in Figure 4 and compared with experimental spectra of Bonsor et al.^25^ recorded for solid (Im)_2_H^+^ClO_4_^–^ and for (Im)_2_H^+^ClO_4_^–^ + Im in equimolar solution (spectra A and B, respectively). In our model calculations we used the calculated value of the ν_s_ frequency as the vertical excitation energy of this mode without tunneling (between nonsplit levels *n*_*x*_ = 0 and *n*_*x*_ = 1). This calculated energy is overestimated mainly due to the harmonic approximation in the *ab initio* vibrational analysis. Promotive and suppressive effects of the low-frequency modes on tunneling produce in our model calculations large numbers of closely spaced sublevels forming four quasi-bands instead of the strict *E*_0_^+^, *E*_0_^–^, *E*_1_^+^, and *E*_1_^–^ levels. In consequence, calculated allowed absorption frequencies, represented by many Dirac δ-peaks, have been presented in Figure 4 as filled rectangles with widths that represent the range of all calculated absorption energies for either the T1 transition or the T2 transition. The oblique projection of the calculated absorption doublet (C in Figure 4) on the experimental spectra (A and B) has been done for clear comparison of the calculated and experimental gap in the absorption doublet. Our simple model relatively well-reproduces the experimentally observed doubling of the absorption band coming from the hydrogen-bonded N–H stretching band in the systems where the hydrogen-bonded ImH^+^–Im complex is formed. It should be mentioned that the two absorption peaks appearing at about 3200 cm^–1^ in spectra A and B come from N–H stretching of non-hydrogen-bonded N–H bonds in imidazole rings (N2–H3 and N2′–H3′ bonds in Figure 2). These two modes are not the subject of our modeling as the N2–H3 and N2′–H3′ groups are not involved in hydrogen bonding and no proton tunneling occurs within these groups. Our results also partly explain the broadening of the doublet components as an effect of the sequence of many transitions occurring at different tunneling splittings being the effect of modulation by low-frequency coupled modes. The broadening effect observed in the IR spectra may be also caused by anharmonic vibrational couplings within the hydrogen bond as well as the interactions in the crystal lattice or in solution. Our model does not reproduce the temperature dependence of the IR absorption bandwidths and evolution of fine structures of the IR absorption bands, as transition intensities are unavailable in our calculations.

**Table 4:** Parameters of the Two-Dimensional Model Potentials Given by Eqs 9 and 10

mode	*x*_0_	α	γ
ν_σ_	2.72	0.215	
ν_β1_	2.79		0.00306
ν_β2_	2.78		0.00311

**Table 5:** Energy Splittings Calculated for the Two-Dimensional Model Potentials for the Ground and the First Excited Vibrational State of the ν_s_ Mode in the ImH^+^–Im Complex

	SMC	SQZ	
quantum no. *n*_*y*_	ν_σ_	ν_β1_	ν_β2_
ν_s_ (*n*_*x*_ = 0)	Δ*E*_0_ (cm^–1^)	Δ*E*_0_ (cm^–1^)	Δ*E*_0_ (cm^–1^)
0	96.5	93.2	92.6
1	99.9	90.5	89.8
2	103.3	87.8	87.1
3	106.7	85.2	84.4
4	110.0	82.6	81.8
5	113.3	80.1	79.2
6	116.6	77.6	76.7
7	119.8	75.2	74.2
8	123.0	72.8	71.8
9	126.1	70.5	69.4
10	129.2	68.2	67.0
ν_s_ (*n*_*x*_ = 1)	Δ*E*_1_ (cm^–1^)	Δ*E*_1_ (cm^–1^)	Δ*E*_1_ (cm^–1^)
0	304.4	317.8	314.5
1	314.5	311.2	307.9
2	324.6	304.6	301.3
3	334.6	298.1	294.8
4	344.6	291.7	288.3
5	354.5	285.4	281.9
6	364.4	279.2	275.5
7	374.2	273.1	269.2
8	384.0	267.1	262.9
9	393.8	261.2	256.7
10	403.6	255.4	250.5

The presented results are important because they allow us to explain in a quantitative way the mechanism of the experimentally observed IR absorption band splitting in the N–H stretching band in the imidazole–imidazolium system in the crystal and in the aqueous solution. We present for the first time the quantitative incorporation of the tunneling effect into the interpretation of the band-doubling and band-broadening effects in the IR absorption spectra in the electronic ground state in the hydrogen-bonded system. The paper presents a model approach that allows us to obtain quantitative results related to the proton tunneling phenomenon in a universal manner which is applicable to many similar systems.

The presented results may be useful for interpretation of infrared spectra of hydrogen-bonded complex cations in crystals as well as in solutions; they can also be a kind of indicator of the presence of such cations in complex systems which have potential applications, such as proton conductors in electrochemical systems used as proton-conducting polyelectrolyte membranes in fuel cells.

## V Conclusions

Proton tunneling dynamics in the hydrogen-bonded imidazole–imidazolium (ImH^+^–Im) complex cation has been studied by performing *ab initio* CASSCF quantum mechanical calculations of the electronic structure for the stable and saddle point geometries, as well as its harmonic vibrational frequencies. On the basis of *ab initio* calculations, two-dimensional model potentials were constructed to take into account the coupling between the high-frequency tunneling mode and the low-frequency hydrogen-bond vibrations affecting the tunnelling process. Tunneling energy splittings for different vibrationally excited states of the low-frequency modes have been calculated for the ground and the first excited state of the tunneling mode on the basis of parametrized model potentials. Within the calculated tunneling splittings an effect of promotion of the tunneling by the excitation of the hydrogen-bond coaxial mode and suppression by the excitation of the bending modes can be observed. On the basis of the calculated tunneling splittings in the ground and the first excited state of the N–H stretching mode, the splittings of the doublet observed in the IR spectra of the ImH^+^–Im complex have been calculated. The experimentally observed N–H stretching IR absorption band doubling has been reproduced qualitatively by our model calculation.

## Notes

The data that support the findings of this study are available from the corresponding author upon reasonable request.
